# It Is Much Better to Reprove Openly Than to Be in Anger Secretly

**Published:** 1874-05

**Authors:** 


*"It is much better to Reprove Openly than to be in Anger Secretly."*

Dr. E.: The most efficient means of getting rid of quacks is to elevate the standard of attainment in the medical colleges. So long as this is low it will be difficult for private members or journalists to accomplish much. Quackery will triumph as long as she sees herself reflected from the colleges. The determination of the members of your Society to make qualification a requisite for fellowship and recognition, in addition to a diploma from a school of character, is a step in the right direction. Stick to it, and it will prove to be true leaven of reform and improvement that will continue to work till the whole mass is leavened. The facilities and means for teaching, and the character of teachers, are of vital importance in this movement of reform. Medical science is rapidly advancing. Colleges should keep pace. If they do not, the time is not far distant when the position you and your Society have taken will be endorsed by all educated professional men, viz: that a DIPLOMA is not *prima facia* evidence of a *professional education.* To accomplish all you desire, students must be educated, medical institutions must have many facilities, teachers must have learning and character. P.

